# ADAPTING THE DETECT TOOL FOR HOME-BASED PRIMARY CARE: INSIGHTS ON POLICIES, PROVIDER PERSPECTIVES, AND TOOL DESIGN

**DOI:** 10.1093/geroni/igae098.2054

**Published:** 2024-12-31

**Authors:** Kristin Lees Haggerty, Brad Cannell, Olanike Ojelabi, Randi Campetti, Jason Burnett, Carolyn Pickering, Melvin Livingston

**Affiliations:** Education Development Center, Waltham, Massachusetts, United States; The University of Texas Health Houston, Houston, Texas, United States; Education Development Center, Waltham, Massachusetts, United States; Education Development Center, Waltham, Massachusetts, United States; The University of Texas Houston, Houston, Texas, United States; The University of Texas Health Houston, Houston, Texas, United States; Emory University, Atlanta, Georgia, United States

## Abstract

Elder mistreatment (EM) is difficult to detect and often goes unrecognized. Home-based primary care (HBPC) providers have enduring relationships with patients who are vulnerable to EM and the ability to see patients’ homes first-hand. Effective and efficient screening tools are urgently needed to support these providers in consistently assessing patients for signs of EM. This project includes a two-stage approach to adapting and rigorously evaluating an evidence-based EM screening tool, Detection of Elder mistreatment Through Emergency Care Technicians (DETECT), for use in home-based primary care (HBPC). In this presentation, we share results from a qualitative study conducted to inform tool adaptation and implementation strategy for DETECT revised for primary care (DETECT-RPC). This study addressed three research questions: 1) what are the implications of state- and site-specific policies on DETECT-RPC implementation, 2) what are the perceived barriers and facilitators to recognizing and reporting EM among HBPC providers, and 3) What adaptations need to be made to make DETECT-RPC feasible and effective in HBPC? Data collection included review of publicly available information and interviews and focus groups with HBPC staff from seven sites (N=16). Interview and focus group data were transcribed and coded using a thematic analysis approach. We will present key themes from the qualitative study including implications for the DETECT-RPC tool design and workflow, and key considerations for developing reporting guidelines structure.

